# Resveratrol Ameliorates Glucocorticoid-Induced Bone Damage in a Zebrafish Model

**DOI:** 10.3389/fphar.2019.00195

**Published:** 2019-03-22

**Authors:** Qun Luo, Shengbo Liu, Liming Xie, Yongjie Yu, Limin Zhou, Yuzhen Feng, De Cai

**Affiliations:** ^1^ Department of Pharmacy, Affiliated Hospital of Guangdong Medical University, Zhanjiang, China; ^2^ Department of Pharmacy, The Central People’s Hospital of Zhanjiang, Zhanjiang, China; ^3^ First Clinical Medical College, Guangdong Medical University, Zhanjiang, China; ^4^ School of Pharmacy, Guangdong Medical University, Zhanjiang, China

**Keywords:** resveratrol, bone mineralization, dexamethasone, bone damage, zebrafish model

## Abstract

Resveratrol (Res) is a multi-functional polyphenol compound that has protective functions in cardiovascular and neurodegenerative diseases. This study aimed to determine the effect of Res on osteogenic differentiation and bone mineralization in zebrafish (*Danio rerio*) with dexamethasone (Dex)-induced bone damage. Our results showed that Dex exposure (15 μmol/l) decreased the green fluorescence areas and the integrated optic density (IOD) values in the skull bones of zebrafish larvae of the TG(SP7:EGFP) strain in a dose-dependent manner (*p* < 0.01). Furthermore, Dex exposure decreased the alizarin red S-stained areas (bone mineralization area) in the skeleton and spinal bones of zebrafish larvae of the AB strain in a dose-dependent manner (*p* < 0.01). By contrast, Res treatment (150 μmol/l) significantly increased both the green fluorescence and bone mineralization area in Dex-exposed zebrafish larvae. Thus, our data show that Res improves bone mineralization after glucocorticoid-induced bone damage in a zebrafish model. Res may be a candidate drug for the prevention of osteoporosis.

## Introduction

Resveratrol (Res; 3,4′,5-trihydroxystilbene) is a multifunctional polyphenol found in the seeds of plants, such as grapes (Polonio et al., 2014), peanuts (Xiong et al., 2014), giant knotweed, and cinnamon (Di Benedetto et al., 2018). Res is a plant antitoxin with antioxidant functions; it is produced in response to pathogens and adverse environmental changes. In nature, Res primarily exists in its glycoside form. It has two geometric isomers: cis and trans, which are usually present in plants with 3β-D2 glucosides. The removal of the glucosyl group from Res *via* hydrolysis leads to the formation of polydatin. In humans, polydatin is degraded by glycosidases in the intestinal tract to release Res.

Res has been found to play a protective role in cardiovascular and neurodegenerative diseases (Wicinski et al., 2017). In the nervous system, Res activates sirtuin 1 protein, whose antioxidant properties reduce β-amyloid protein production and protect neuronal cells from β-amyloid protein-induced free radical-mediated DNA damage (Deng and Mi, 2016). Res plays a similar role in yeast, by limiting energy uptake and activating the key yeast longevity gene *Sir2*, which mediates deacetylation and delays aging (Orlandi et al., 2017). Additionally, Res has antitumor activities and promotes osteoblast differentiation (Di Benedetto et al., 2018). It inhibits the proliferation of chondrosarcoma cells and the proliferation and phosphorylation of components in the STAT3 signaling pathway and induces tumor cell apoptosis (Zhang et al., 2015). Thus, Res may have extensive biological applications; for example, as Res promotes osteogenic differentiation and estrogen-like functions, it may serve as an effective treatment for osteoporosis (Tseng et al., 2011; Di Benedetto et al., 2018). However, the precise role of Res in osteogenic differentiation is still not clear.

We therefore designed a study to investigate the role of Res in a zebrafish (*Danio rerio*) bone damage model. The genomes of zebrafish and mammals share high homology, with similar genome- and protein regulation mechanisms. Zebrafish models have the advantages of small individual volume (appropriate for high-throughput drug screening) (Barrett et al., 2006), short reproductive cycle, strong reproductive ability, ease of maintenance, external fertilization, rapid development, and transparent embryos (convenient for the observation of skeletal development). Therefore, zebrafish is extensively used in the fields of drug screening, toxicity detection, and development research. The bones of zebrafish larvae have cells that are required for bone formation and bone resorption, and a zebrafish model of glucocorticoid-induced osteoporosis (GIOP) has already been established (Barrett et al., 2006; Schaaf et al., 2009). This model allows the observation of bone development in the zebrafish head and has been demonstrated to be helpful for screening drugs that promote bone development (Su et al., 2016).

The aim of this study was to determine the usefulness of Res in counteracting glucocorticoid-induced bone damage and mortality in a zebrafish model. We also analyzed the effects of Res on bone mineralization, bone formation, and osteogenic differentiation in zebrafish. We hope to provide experimental evidence of the biological effects of Res in the prevention and treatment of GIOP, and establish a foundation for future studies on the mechanism(s) of action of Res in osteoporosis.

## Materials and Methods

### Experimental Animals

Adult zebrafish [TG(SP7:EGFP) strain] were provided by the Chung Yuan Christian University (Taiwan), and the feeding laboratory was provided by the Zebrafish Model Animal Base of the Affiliated Hospital of Guangdong Medical University. Adult wild-type AB-strain zebrafish and the transgenic TG(SP7:EGFP) strain were maintained under a 14 h:10 h day/night cycle. Natural mating was allowed to occur between the following pairs of wild-type AB-strain zebrafish and TG(SP7:EGFP)-strain zebrafish (DeLaurier et al., 2010; Knopf et al., 2011): male and female TG zebrafish, male TG and female AB-strain zebrafish, female TG and male AB-strain zebrafish, and male and female wild-type AB-strain zebrafish. The fertilized zebrafish eggs thus produced were collected and placed in cell-culture dishes. The eggs were incubated in egg water (5 mmol/l NaCl, 0.17 mmol/l KCl, 0.33 mmol/l CaCl_2_, 0.33 mmol/l MgSO_4_, and 0.00001% methylene blue) in a thermostat incubator at 28.5 ± 0.5°C. The culture medium was replaced with fresh culture medium (egg water) containing 1-phenyl-2-thiourea. After 36 h of fertilization, embryo identification was performed using fluorescence microscopy.

### Reagents and Instruments

We purchased dexamethasone sodium phosphate (Dex) injection solution (H42020019, ≥97%) from Tianjin Jinyao Group, Hubei Tianyao Pharmaceutical Industry Co. Ltd.; Res from Sigma (USA, CAS no.: 501-36-0; ≥99%, 1002179582); alizarin red S (100375) and dimethyl sulfoxide (DMSO, 196055) from MP Biomedicals; low-melting-point agarose from Life Technologies (16520-100); methylcellulose from Sigma (1001777023); Tween-20 from Guangdong Guanghua Sci-Tech Co. Ltd. (9005-64-5); anhydrous ethanol from Shanghai Sangon Biotech (ET0737); and methanol from Tianjin Damao Chemical Reagent Factory (67-56-1).

We used a SZX10 fluorescence microscope from Olympus (Germany), a Leica fluorescence microscope-imaging system (M2057A) from Leica (Germany), the Image Pro Plus 6.0 professional image analysis software from Media Cybernetics (USA), and a thermostat incubator.

### Drug Preparation

Dex was dissolved in DMSO to prepare a 1,000 μmol/l stock solution. The stock solution was then serially diluted in egg water to obtain solutions with Dex concentrations of 1, 2.5, 10, 15, 20, and 25 μmol/l. Similarly, a 250 μmol/l Res stock solution was prepared. This solution was mixed with DMSO and serially diluted with the culture medium (egg water) to obtain solutions with Res concentrations of 25, 50, 75, 100, 150, 200, and 250 μmol/l. To prepare solutions containing both Res and Dex, we added 15 ml of the Dex stock solution (1,000 μmol/l) to an appropriate volume of the Res stock solution (250 μg/ml) to obtain a final Dex concentration of 15 μmol/l. This solution was then diluted to obtain the Res concentrations shown above. Each of these Res solutions contained 15 μmol/l Dex and 0.5% DMSO.

### Induction of Bone Damage in Zebrafish Using Dex

At 3 days post-fertilization (dpf), the TG(SP7:EGFP)-strain zebrafish and the AB-strain zebrafish embryos were examined under a fluorescence microscope. Embryos from the wild-type AB-strain pairs were also screened for fluorescence to determine whether the offspring of the wild-type pairs showed green fluorescence or any other fluorescence. Embryos that showed green fluorescence were transferred to two 24-well plates (4 × 6) to each well of which, 1 ml culture medium was added. Five randomly selected larvae were placed in each well. The larvae were divided into a negative-control group and several Dex groups (0.25, 1.00, 5.00, 10.00, 15.00, 20.00, and 25.00 μmol/l). For each concentration, five groups of samples were tested. Each group contained five wells, and each well contained five fish. At 4 dpf, the culture medium in each well of the 24-well plate was aspirated as well as possible and immediately replaced with the Dex solutions prepared using the culture medium (0.25, 1.00, 5.00, 10.00, 15.00, 20.00, and 25.00 μmol/l Dex with 0.5% DMSO). The plate was then further incubated in a thermostat incubator at 28.5 ± 0.5°C. The solution in each well was aspirated using a pipette every other day, and an equal volume of new solution was added. The culture cycle was 6 days (9 dpf). The zebrafish larvae were not fed during the culture process. At 9 dpf,through the Image, we selected 15 fish with less differences among individuals from 5 groups of fish for testing.

### Inhibition of Bone Damage in Zebrafish Using Res

The zebrafish feeding and grouping methods in this experimental step were consistent with those described above. We used several concentrations of Res (25, 50, 75, 100, 150, 200, and 250 μmol/l) to treat Dex-induced bone damage in the zebrafish. Res was administered between 3 and 9 dpf, and mortality rates were calculated during the same period.

### Detection of Fluorescence in Transgenic Bone of TG(SP7:EGFP)-Strain Zebrafish

At 9 dpf, the cultured TG(SP7:EGFP)-strain larvae were transferred to MS-222 anesthetic solution (10 mg/l). The anesthetic solution was removed, and the larvae were embedded in 1% low-melting-point agarose. The position of the larvae was adjusted such that the left lateral surface was visible from above. A Leica M205 stereoscopic fluorescence microscope was used to observe the green fluorescent areas in the left side of the skull (seen from the back and distributed symmetrically on the left and right sides), including the operculum system (operculum + branchial arch), mandible, and maxilla. Only hard bones could be detected; the cartilage did not fluoresce. The fluorescent photos were numeralized using Image J software, which transformed the images into gray-scale images at eight-bit precision. Selected areas were analyzed to determine their surface area; mean, minimum, and maximum gray-scale values; and integrated optical density (IOD). A DFC 310 FX camera (Leica, Germany) was used to capture the fluorescent images (100×). All images were obtained using the same light intensity and exposure settings (Luo et al., 2016). The sample size of each group was *n* = 15.

### Alizarin Red S Staining and Quantitative Analyses of Bone Mineralization of Zebrafish Skeleton

AB-strain zebrafish cultured to 9 dpf were euthanized by placing them in MS-222 anesthetic agent (100 mg/l). The anesthetic agent solution was removed, and the larvae were fixed with 4% paraformaldehyde solution for 2–2.5 h. The paraformaldehyde solution was removed, and the larvae were stained with the alizarin red S staining solution (Walker and Kimmel, 2007). Under the Leica M205 stereoscopic fluorescence microscope, red-stained bones were observed in the operculum, branchial arch, mandible, and maxilla and in the branchial system, mandible, maxilla, and vertebrae. Images (100×) were captured using the DFC 310 FX camera (Leica, Germany). All images used consistent light intensity and exposure settings (Luo et al., 2016). The Image Pro Plus 6.0 software was used to calculate the surface area and IOD of the alizarin red-stained regions (the eyes, otolith, and pigment residues were excluded). The bone mineralization level and bone density were determined (IOD was area multiply by mean gray value). The sample size of each treatment group was *n* = 15.

To assess the repeatability of the microscopic analysis of the zebrafish skeleton, the zebrafish larvae skeletons were transferred onto new slides after the above analysis. Their position was readjusted, and stained images were again collected. This procedure was performed three times.

### Statistical Analysis

Image Pro Plus v6.0 software was used to calculate the surface area and IOD of the fluorescent and alizarin red S-stained regions. The data were analyzed using SPSS v22.0. One-way analysis of variance ANOVA was performed to determine the normality of all data. Differences with *p* less than 0.05 were considered statistically significant. All parameters were presented as mean ± SD.

## Results

### The Size of Zebrafish

The sample size of 15 TG(SP7:EGFP) zebrafish were selected from each group. The Image was used to calculate the lateral surface area of the zebrafish. The results showed that there was no significant difference in the size of each fish compared with each group (*p* > 0.05; Tables 1 and 2).

**Table 1:** The lateral surface area of zebrafish bone damage induced by Dex ($\bar{\chi }\pm S$, *n* = 15).

Concentration	Lateral surface area	*p* *
Control (dimethyl sulfoxide)	143,040.33 ± 1,257.366	–
Dexamethasone (Dex) (μmol/l)		
0.25	143,111.87 ± 2,420.260	0.922
1.00	143,559.67 ± 1,292.906	0.478
5.00	143,481.93 ± 4,014.284	0.547
10.00	143,594.87 ± 1,869.145	0.449
15.00	143,524.00 ± 1,077.477	0.509
20.00	143,586.07 ± 1,185.661	0.456
25.00	143,645.87 ± 1,037.944	0.409
30.00	143,001.47 ± 1,908.75	0.958

* Compared with control.

**Table 2:** Lateral surface area of Res *vs.* 15 μmol/l Dex induced bone damage in zebrafish ($\bar{\chi }\pm S$, *n* = 15).

Concentration	Lateral surface area	*p* ^*^	*p* ^#^
Control (dimethyl sulfoxide)	140,529.47 ± 2,385.748	–	0.224
Resveratrol (Res) (μmol/l)			
25.00	139,465.47 ± 3,981.309	0.263	0.922
50.00	139,200.20 ± 3,983.320	0.163	0.856
75.00	139,980.07 ± 2,704.507	0.563	0.522
100.00	139,858.93 ± 1,263.063	0.480	0.609
150.00	139,877.47 ± 2,107.661	0.492	0.595
200.00	139,197.40 ± 2,264.456	0.162	0.853
250.00	139,486.27 ± 1,679.034	0.273	0.905
Dex	139,372.87 ± 1,343.122	0.224	

* Compared with control.

# Compared with model.

### Effect of Dex on Bone Formation and Bone Mineralization

**Figure 1:**
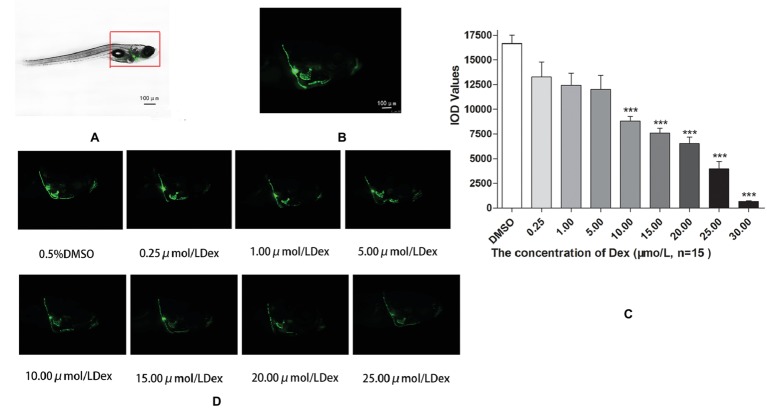
Influence of dexamethasone (Dex) concentration on zebrafish skull. Schematic diagram of TG(SP7:EGFP) zebrafish larvae **(A,B)**. IOD values of green fluorescence of Dex-induced bone damage in TG zebrafish larvae **(C)**. Images of green fluorescence in TG zebrafish larvae skull [in profile **(D)**]. The Dex concentrations used were 0, 0.25, 5.00, 10.00, 15.00, 20.00, and 25.00 μmol/l, and 0.1% DMSO was used as the control. *n* = 15, ***p < 0.01.

**Figure 2:**
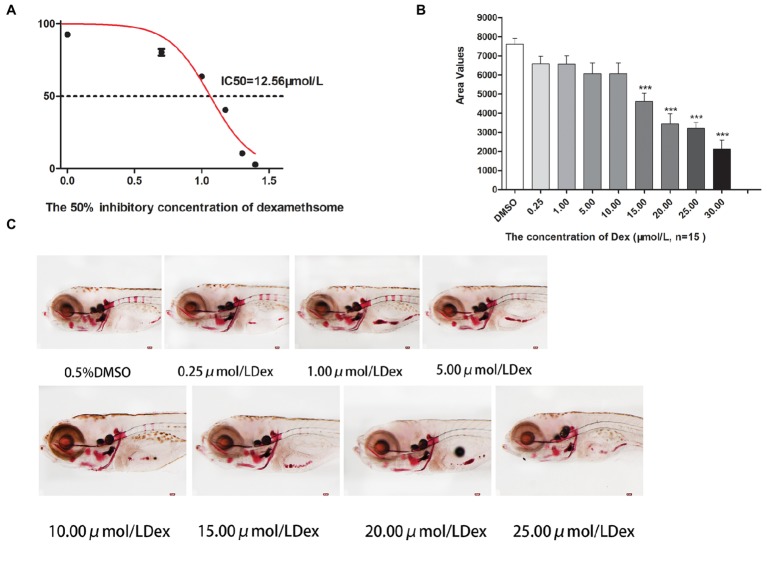
IC_50_ and area of bone mineralization after Dex-induced bone damage in AB-strain zebrafish larvae. The 50% inhibitory concentration of dexamethasone (Dex) **(A)**. Analysis of mineralization area in the skull and spine of Dex-treated AB-strain zebrafish larvae at 9 dpf **(B)**. Alizarin red S staining of the skull in Dex-treated AB-strain zebrafish larvae at 9 dpf **(C)**. The Dex concentrations used were 0.25, 1.00, 5.00, 10.00, 15.00, 20.00, and 25.00 μmol/l. The zebrafish larvae were exposed to Dex from 3 to 9 dpf, and 0.1% DMSO served as the control. *n* = 15, *p < 0.05, ***p < 0.01.

In the TG(SP7:EGFP) zebrafish, the surface area and IOD of the green fluorescent regions, which represented osteoblast differentiation, were significantly lower in the Dex-treated groups than in the control group. Furthermore, as the Dex concentration increased, the values of these two parameters decreased in a dose-dependent manner (*p <* 0.01; Figure 1; Table 3). In the AB-strain zebrafish, the surface area and IOD of the alizarin red-stained areas, which represented the area of skull mineralization, decreased when the Dex concentration increased from 10 to 25 μmol/l (*p <* 0.05; Figure 2; Table 4). These results indicate that Dex reduced bone mineralization and inhibited osteogenic differentiation in zebrafish larvae in a dose-dependent manner. The 50% inhibiting concentration of Dex for the induction of bone damage was 11.56 μmol/l (Figure 2). Higher Dex concentrations (20 and 25 μmol/l) significantly inhibited zebrafish vitality and increased mortality. Therefore, we selected the 15 μmol/l concentration for inducing bone damage in zebrafish in subsequent experiments.

**Table 3:** Integrated optical density values of the green fluorescent area in zebrafish with Dex-induced bone damage ($\bar{\chi }\pm S$, *n* = 15).

Concentration	Integrated optical density	*p* ^*^
Control (dimethyl sulfoxide)	16,660.69 ± 3,198.12	–
Dexamethasone (μmol/l)		
0.25	13,263.82 ± 5,627.98	0.800
1.00	12,421.28 ± 4,646.26	0.249
5.00	12,015.75 ± 5,298.27	0.257
10.00	8,827.41 ± 1,654.84	0.000
15.00	7,588.33 ± 1,692.85	0.000
20.00	6,535.59 ± 2,298.01	0.000
25.00	3,976.45 ± 2,698.18	0.000
30.00	662.92 ± 171.94	0.000

* Compared with control.

**Table 4:** Area of bone mineralization in zebrafish with dexamethasone (Dex)-induced bone damage ($\bar{\chi }\pm S$, *n* = 15).

Concentration	Area of alizarin red S staining	*p* ^*^
Control (dimethyl sulfoxide)	7,612.15 ± 1,113.74	–
Dexamethasone (μmol/l)		
0.25	6,587.21 ± 1,461.87	0.742
1.00	6,573.14 ± 1,604.13	0.802
5.00	6,056.21 ± 2,140.83	0.504
10.00	6,057.85 ± 2,059.75	0.506
15.00	4,621.31 ± 1,520.41	0.000
20.00	3,430.85 ± 1,957.05	0.000
25.00	3,213.92 ± 1,109.91	0.000
30.00	2,117.50 ± 963.52	0.002

* Compared with control.

### Effect of Res on Dex-Induced Bone Damage

**Figure 3:**
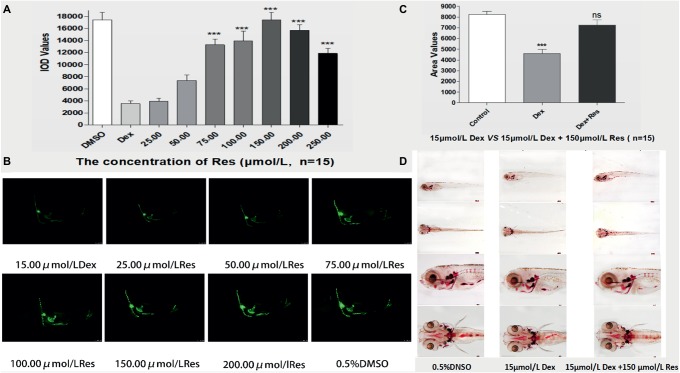
Effect of Res on Dex-induced bone damage in zebrafish. IOD values of green fluorescence of Res after Dex-induced bone damage in TG zebrafish larvae **(A)**. Images of green fluorescence in TG zebrafish larvae skull [in profile **(B)**]. The Res concentrations used were 25.00, 50.00, 75.00, 100.00, 150.00, 200.00, and 250.00 μmol/l, and 0.1% DMSO served as the control. The area of bone mineralization after Res treatment of Dex-induced bone damage in AB-strain zebrafish larvae at 9 dpf [**(C)** 15.00 μmol/l Dex *vs.* 15.00 μmol/l Dex plus 150.00 μmol/l Res]. Alizarin red S staining of the skull after Res treatment of Dex-induced bone damage in AB-strain zebrafish larvae at 9 dpf **(D)**. *n* = 15, *p < 0.05, ***p < 0.01.

Compared to the Dex groups, the Res-treated groups showed significantly increased IOD of green fluorescence in TG(SP7:EGFP) zebrafish bones and increased bone mineralization area in the AB-strain zebrafish bones after exposure to 15 μmol/l Dex (*p <* 0.01; Figure 3; Tables 5 and 6). As the Res concentration increased, its effect on counteracting the bone damage induced by 15 μmol/l Dex also increased. A Res concentration of 150 μmol/l significantly counteracted the bone damage induced by 15 μmol/l Dex.

**Table 5:** Integrated optical density values of green fluorescence in zebrafish treated with resveratrol (Res) and dexamethasone (Dex) ($\bar{\chi }\pm S$, *n* = 15).

Concentration	Integrated optical density	*p* ^*^
Control (15 μmol/l dexamethasone)	3,539.01 ± 1,957.47	–
Resveratrol (μmol/l)		
25.00	3,894.76 ± 2,262.19	1.000
50.00	7,352.80 ± 2,938.60	0.068
75.00	13,282.34 ± 5,148.00	0.000
100.00	13,946.22 ± 7,711.83	0.000
150.00	17,394.34 ± 4,989.93	0.000
200.00	15,660.99 ± 4,141.60	0.000
250.00	12,500.61 ± 2,717.08	0.000
Dimethyl sulfoxide	17,420.11 ± 5,880.07	0.000

* Compared with control.

**Table 6:** Area of bone mineralization in the skull and spine of zebrafish treated with resveratrol (Res) and dexamethasone (Dex) ($\bar{\chi }\pm S$, *n* = 15).

Concentration	Area of alizarin red S staining	*p* ^*^
Control (dimethyl sulfoxide)	8,106.17 ± 1,049.75	–
15 μmol/l dexamethasone	4,600.46 ± 1,442.27	0.000
15 μmol/l dexamethasone + 100 μmol/l Resveratrol	6,858.59 ± 1,954.06	0.051

* Compared with control.

### Effect of Res on Zebrafish Survival

**Figure 4:**
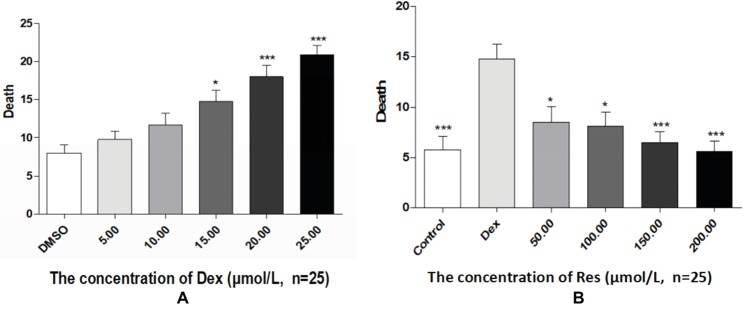
Comparison of survival rates of zebrafish exposed to Dex and Res. Survival rate of zebrafish larvae with Dex-induced bone damage **(A)**. Survival rate of zebrafish larvae with Dex-induced bone damage treated using Res **(B)**. *n* = 25, *p < 0.05, ***p < 0.01.

Compared with the DMSO group, the Dex groups showed higher zebrafish mortality rates, which increased with an increase in Dex concentration, in a dose-dependent manner. The 20 and 25 μmol/l concentrations of Dex induced significant mortality. Compared with the Dex group, the Res groups showed significantly lower mortality. As the Res concentration increased, its effect on counteracting Dex (20 μmol/l)-induced zebrafish death significantly increased (*p* < 0.01; Figure 4; Tables 7 and 8).

**Table 7:** Survival rates of dexamethasone (Dex)-treated zebrafish (*n* = 25).

Concentration	Survival rate (mean ± SD)	Death rate (mean ± SD)	*p* ^*^
Control (dimethyl sulfoxide)	0.68 ± 0.058	0.32 ± 0.058	–
Dexamethasone (μmol/l)			
5.00	0.61 ± 0.061	0.39 ± 0.061	0.967
10.00	0.53 ± 0.062	0.47 ± 0.062	0.567
15.00	0.41 ± 0.061	0.59 ± 0.061	0.024
20.00	0.28 ± 0.056	0.72 ± 0.056	0.001
25.00	0.16 ± 0.046	0.84 ± 0.046	0.000

* Compared with control.

**Table 8:** Survival rate of zebrafish exposed to resveratrol (Res) and dexamethasone (Dex) (*n* = 25).

Concentration	Survival rate (mean ± SD)	Death rate (mean ± SD)	*p* ^*^
Control (20 μmol/l dexamethasone)	0.04 ± 0.061	0.59 ± 0.061	–
Resveratrol (μmol/l)			
50.00	0.66 ± 0.059	0.34 ± 0.059	0.117
100.00	0.68 ± 0.058	0.32 ± 0.058	0.053
150.00	0.74 ± 0.054	0.26 ± 0.054	0.004
200.00	0.78 ± 0.052	0.22 ± 0.052	0.002
Dimethyl sulfoxide	0.77 ± 0.053	0.23 ± 0.053	0.005

* Compared with control.

## Discussion

**Figure 5:**
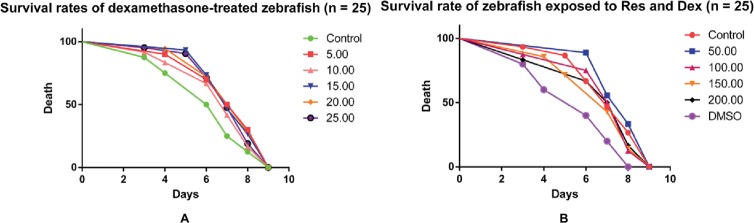
The curves of survival rates of zebrafish exposed to Dex and Res. Survival rates of dexamethasone-treated zebrafish **(A)**; Survival rate of zebrafish exposed to resveratrol and dexamethasone **(B)**.

The indicators observed in this study were the IOD value of green fluorescent protein (GFP) fluorescence in the TG zebrafish and the area of alizarin red S staining in the bones of the AB-strain zebrafish. In addition, the effect of Res on the survival of Dex-treated zebrafish was observed (Figure 5). TG(SP7:EGFP) is a strain of transgenic zebrafish that expresses GFP driven by the Osterix gene, which is involved in osteogenic differentiation (DeLaurier et al., 2010). The osteoblasts of TG(SP7:EGFP) are selectively labeled with GFP; therefore, the IOD value of the local green fluorescence directly reflects the differentiation and number of osteoblasts. Higher expression levels in the TG strain indicate higher osteogenic differentiation levels. Osteoblast differentiation and bone formation can be visualized in the TG larvae by monitoring the changes in GFP fluorescence intensity under a fluorescence microscope. Alizarin red S is a special stain used for bone staining. This stain can be used to determine the bone mineralization level based on the color, and the area of alizarin red S staining can be used to determine efficacy of drugs affecting bone mineralization. Bone mesenchymal stem cells have a strong self-proliferation ability and multidirectional differentiation potential. They are induced by different inducers to differentiate into progenitor cells of various types of cells, such as fibroblasts, adipocytes, chondrocytes, and osteoblasts. We have previously shown that Res promotes the differentiation of bone mesenchymal stem cells into osteoblasts and increases the activities of alkaline phosphatase and prolyl hydroxylase in a dose-dependent manner (De Cai et al., 2014).

Our results indicated that Dex exposure inhibited osteogenic differentiation and bone mineralization in zebrafish larvae in a dose-dependent manner (*p* < 0.05; Figure 1; Table 1). We found that exposure to 15 μmol/l Dex significantly inhibited bone formation and mineralization in the zebrafish larvae (*p* < 0.05). Bone mineralization is a biochemical process in which inorganic minerals are deposited in organic bone. In physiological conditions, calcium salts dissolve and deposit in bone tissue and maintain a dynamic balance under the interaction of osteoblasts and osteoclasts. In pathological conditions, this dynamic balance is impaired, and bone resorption exceeds bone formation, resulting in defective bone mineralization. This results in trabecular bone destruction, reduced bone volume, decreased bone strength, increased brittleness, and ultimately osteoporosis (Guangwen et al., 2016). Glucocorticoids directly affect osteoclasts, osteoblasts, and bone cells. They reduce osteoblast production, promote osteoblast and bone-cell apoptosis, prolong osteoclast life, and weaken the bone, thereby leading to osteoporosis. Indeed, long-term glucocorticoid use may cause GIOP, which is a major side effect and one of the most common types of secondary osteoporosis (Popp et al., 2006).

This study showed that Res significantly increased bone formation and mineralization in Dex-treated zebrafish (*p* < 0.05). The optimum Res concentration was 150 μmol/l. Additionally, Res significantly increased the survival of Dex-treated zebrafish (*p* < 0.01). It has been suggested that Res can increase bone mineralization, promote osteoblast formation and osteogenic differentiation, enhance bone strength, and effectively resist Dex-induced bone damage (Shakibaei et al., 2012; Ma et al., 2018). Res is a non-flavonoid polyphenol compound and a phytoestrogen. Because its chemical structure is similar to that of estrogen, Res has estrogen-like activity and bone-protective functions. It can compete with 17 isoyl-estradiol to bind estrogen receptors *in vitro* and activate the transcription of estrogen-responsive genes (Gehm et al., 1997). In one study, bone density was found to increase in postmenopausal women who drank about four glasses of wine a day (Bertelli et al., 1996). γ-Glutamine synthase is a catalytic enzyme involved in the synthesis of glutathione, which can resist oxidative stress and maintain the redox balance *in vivo* (Rahman and MacNee, 2000). Heme oxygenase-1 can catalyze hemoglobin to decompose oxygen molecules and reduce the production of reactive oxygen species (ROS). Other antioxidant enzymes, such as catalase, can also be upregulated to enhance antioxidant capacity (Barbagallo et al., 2010; Castro-Alférez et al., 2017). Zhang et al. (Tiejun et al., 2012) showed that Res can improve the activities of the antioxidant enzymes in bone tissue, and thereby reduce oxidative stress, maintain metabolic balance, and prevent bone-mass loss. Additionally, Res has been shown to improve the osteogenic capacity of dental bud stem cells, and its glucoside polydatin has been shown to enhance the osteogenic differentiation of these cells (Di Benedetto et al., 2018).

Epidemiological and genetic studies of humans and rodents have indicated that ROS accumulation and increased oxidative stress play critical roles in the development of osteoporosis (Lee et al., 2015; Sharma et al., 2015). In bone tissues, excessive ROS production directly promotes osteoblast and bone-cell apoptosis (Sato et al., 2015). Additionally, ROS-induced oxidative stress increases osteoclast differentiation (Shi et al., 2015). Some studies have indicated that excessive glucocorticoids increase ROS levels in the skeletal muscles and thereby induce oxidative stress (Feng and Tang, 2014). Therefore, it is reasonable to hypothesize that antioxidant therapy may counteract osteoporosis.

In conclusion, our study shows that Res significantly counteracts glucocorticoid-induced bone damage in zebrafish. Res may serve as a natural estrogen that can prevent and treat osteoporosis by counteracting the effects of glucocorticoid-induced increases in ROS production and oxidative stress. It may effectively promote osteoblast formation, bone mineralization, and osteogenic differentiation to protect bone cells and maintain a balance between bone formation and resorption. Currently, the mechanisms underlying the Res-induced promotion of osteogenic differentiation in zebrafish are not completely clear and warrant further study. To further elucidate these mechanisms, we intend to investigate the effect of Res on the expression of various key target genes, transcriptional factors, and signaling pathways involved in osteogenesis, such as the bone sialoprotein, osteocalcin, Osterix, sclerostin, cathepsin K, Wnt, and OPG/RANKL pathways, in a zebrafish model of GIOP in the future. The present results have the potential to establish a clear scientific basis for exploiting Res as a candidate drug for osteoporosis treatment.

## Ethics Statement

This study was carried out in accordance with the recommendations of ‘Experimental Animals Committee of Guangdong Medical University.” The protocol was approved by the “Experimental Animals Committee of Guangdong Medical University.”

## Author Contributions

DC and QL were the lead curators on this project. QL and SL wrote the manuscript. YF and LX performed the microscopy and analysis. SL, YY, and LZ provided assistance with zebrafish breeding and analysis.

### Conflict of Interest Statement

The authors declare that the research was conducted in the absence of any commercial or financial relationships that could be construed as a potential conflict of interest.

## Abbreviations

Dex: Dexamethasone
DMSO: Dimethyl sulfoxide
Dpf: Days post-fertilization
GFP: Green fluorescent protein
GIOP: Glucocorticoid-induced osteoporosis
IOD: Integrated optical density
Res: Resveratrol
ROS: Reactive oxygen species.
