# Corrigendum: Resveratrol Ameliorates Glucocorticoid-Induced Bone Damage in a Zebrafish Model

**DOI:** 10.3389/fphar.2019.00736

**Published:** 2019-07-02

**Authors:** Qun Luo, Shengbo Liu, Liming Xie, Yongjie Yu, Limin Zhou, Yuzhen Feng, De Cai

**Affiliations:** ^1^Department of Pharmacy, Affiliated Hospital of Guangdong Medical University, Zhanjiang, China; ^2^Department of Pharmacy, The Central People’s Hospital of Zhanjiang, Zhanjiang, China; ^3^First Clinical Medical College, Guangdong Medical University, Zhanjiang, China; ^4^School of Pharmacy, Guangdong Medical University, Zhanjiang, China

**Keywords:** resveratrol, bone mineralization, dexamethasone, bone damage, zebrafish model

In the published article, there was a mistake in **Figure 3(D)** and **Figure 4** as published. The corrected **Figure 3(D)** and **Figure 4** appear below. The authors apologize for this error and state that this does not change the scientific conclusions of the article in any way. The original article has been updated.

**Figure 3(D)****:** The picture is correct, but the following explanatory note needed to be added under the picture.

0.5%DNSO            15*μ*mol/L Dex            15*μ*mol/L Dex +150 *μ*mol/L Res

**Figure 3:**
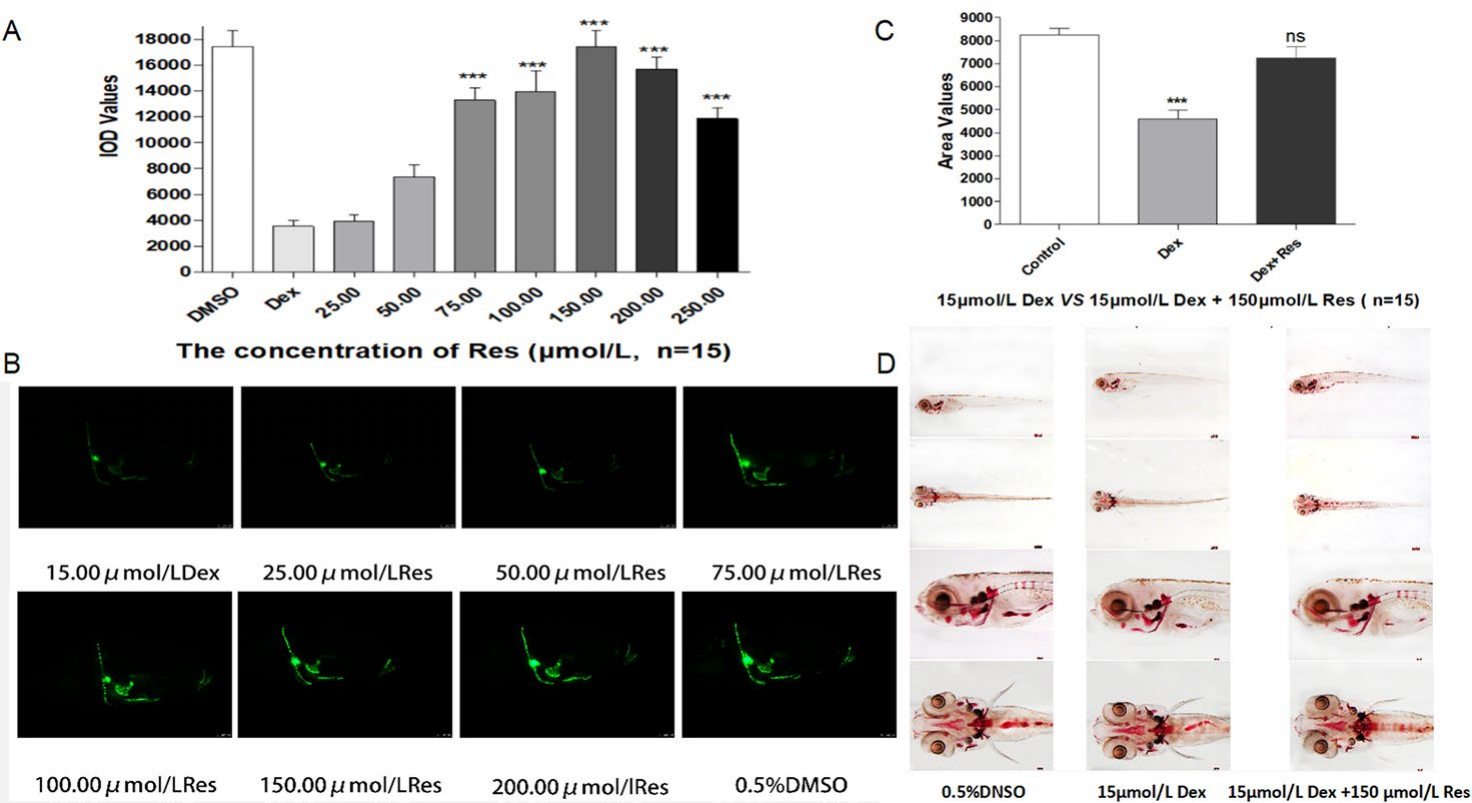
Effect of Res on Dex-induced bone damage in zebrafish. IOD values of green fluorescence of Res after Dex-induced bone damage in TG zebrafish larvae **(A)**. Images of green fluorescence in TG zebrafish larvae skull [in profile **(B)**]. The Res concentrations used were 25.00, 50.00, 75.00, 100.00, 150.00, 200.00, and 250.00 μmol/l, and 0.1% DMSO served as the control. The area of bone mineralization after Res treatment of Dex-induced bone damage in AB-strain zebrafish larvae at 9 dpf [**(C)** 15.00 μmol/l Dex vs. 15.00 μmol/l Dex plus 150.00 μmol/l Res]. Alizarin red S staining of the skull after Res treatment of Dex-induced bone damage in AB-strain zebrafish larvae at 9 dpf **(D)**. *n* = 15, *p < 0.05, ***p < 0.01.

**Figure 4****:** The picture is correct, but the correct explanatory note needed to be added under the picture.

**Figure 4:**
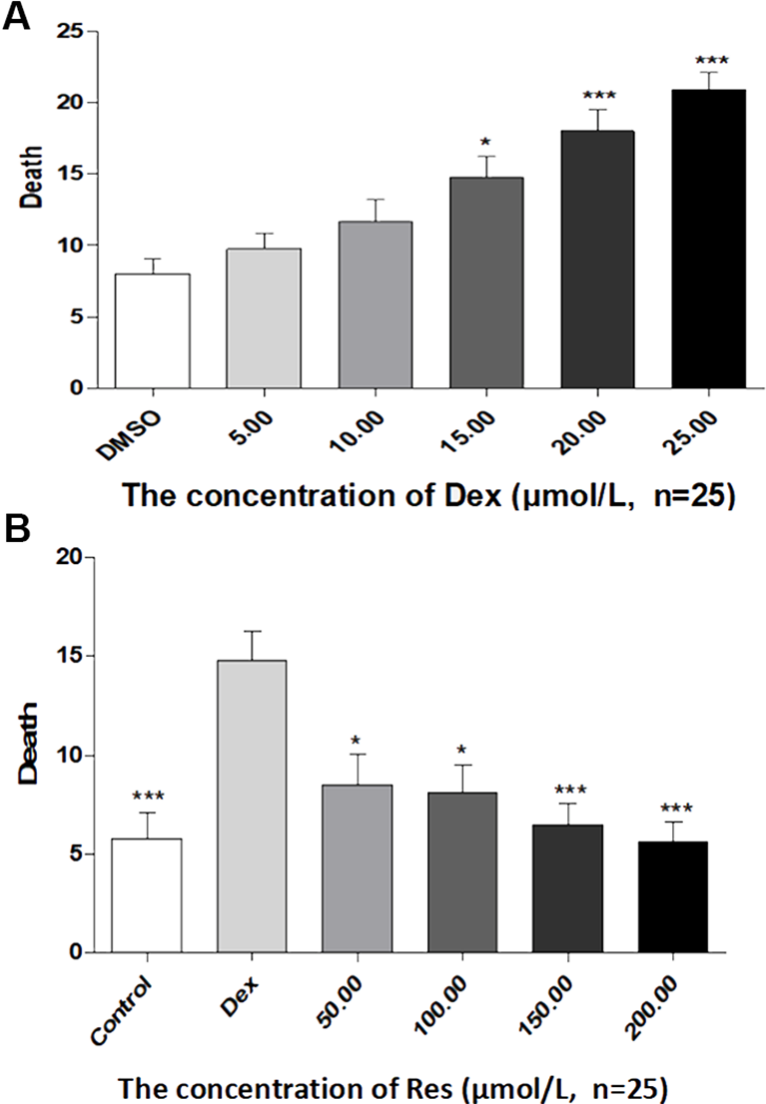
Comparison of survival rates of zebrafish exposed to Dex and Res. Survival rate of zebrafish larvae with Dex-induced bone damage **(A)**. Survival rate of zebrafish larvae with Dex-induced bone damage treated using Res **(B)**. *n* = 25, *p < 0.05, ***p < 0.01.

